# The Impact of Different Kernel Functions on the Performance of Scintillation Detection Based on Support Vector Machines †

**DOI:** 10.3390/s19235219

**Published:** 2019-11-28

**Authors:** Caner Savas, Fabio Dovis

**Affiliations:** Departments of Electronics and Telecommunications, Politecnico di Torino, 10129 Torino, Italy; fabio.dovis@polito.it

**Keywords:** GNSS, scintillation, support vector machines, kernel, Gaussian, polynomial

## Abstract

Scintillation caused by the electron density irregularities in the ionospheric plasma leads to rapid fluctuations in the amplitude and phase of the Global Navigation Satellite Systems (GNSS) signals. Ionospheric scintillation severely degrades the performance of the GNSS receiver in the signal acquisition, tracking, and positioning. By utilizing the GNSS signals, detecting and monitoring the scintillation effects to decrease the effect of the disturbing signals have gained importance, and machine learning-based algorithms have been started to be applied for the detection. In this paper, the performance of Support Vector Machines (SVM) for scintillation detection is discussed. The effect of the different kernel functions, namely, linear, Gaussian, and polynomial, on the performance of the SVM algorithm is analyzed. Performance is statistically assessed in terms of probabilities of detection and false alarm of the scintillation event. Real GNSS signals that are affected by significant phase and amplitude scintillation effect, collected at the South African Antarctic research base SANAE IV and Hanoi, Vietnam have been used in this study. This paper questions how to select a suitable kernel function by analyzing the data preparation, cross-validation, and experimental test stages of the SVM-based process for scintillation detection. It has been observed that the overall accuracy of fine Gaussian SVM outperforms the linear, which has the lowest complexity and running time. Moreover, the third-order polynomial kernel provides improved performance compared to linear, coarse, and medium Gaussian kernel SVMs, but it comes with a cost of increased complexity and running time.

## 1. Introduction

Trans-ionospheric communication of the radio waves while traveling from transmitter to user is affected by the ionosphere that is highly variable and dynamic in both time and space [1]. The ionosphere, highly varied propagation medium, has an irregular structure due to plasma instabilities and scintillation is basically random fluctuations of the parameters of trans-ionospheric waves. Observation of the scintillation has been used in many research fields such as astronomy, geophysics, atmospheric physics, ocean acoustics, telecommunications, etc. so as to identify the irregular structure of the propagation medium [1]. Moreover, it has been found that the atmospheric structure has the basic characteristics of fluid turbulence in the equilibrium range, which is characterized by the well-known properties of Kolmogorov turbulence [2]. It has led to the development of the theoretical characterization of the scintillation effect [3,4] besides the observational point of view of the scintillation effect. However, although scintillation phenomena are well understood and widely exploited, it is difficult to find a definitive treatment of the theory of scintillation [2], and it is a fact of life for a number of communication and radar systems that have to operate through the auroral or equatorial ionosphere [5].

Global Navigation Satellite Systems (GNSS) signals also undergo severe propagation effects such as phase shifts and amplitude variations while they propagate through the Earth’s upper atmosphere, the ionosphere [6]. Ionospheric irregularities affect the GNSS signals in two ways, and both refractive and diffractive effects are gathered under the name of scintillation since they cause large-scale variations in both signal power and phase [7]. Ionospheric scintillation severely may degrade GNSS receiver performance by causing signal power loss and increasing measurement noise level, causing loss of lock of the tracking of GNSS signals [8,9]. In case of strong amplitude and phase scintillation events, it has been observed that even acquisition of the signals can be prevented [10].

Under a scintillation event, proper countermeasures could be undertaken at signal processing level, enabling either more robust signal acquisition and tracking or alternate resources to decrease the effect of disturbed signal propagations. Therefore, detecting and monitoring the scintillation effects in order to estimate the ionospheric scintillation in its early stages and measure the scintillation parameters gains importance. In this sense, GNSS signals provide an excellent means for measuring scintillation effects due to the fact that they are available all the time and can be acquired through many points of the ionosphere simultaneously [11]. With the coming new GNSS systems, a greater number of signals are available for monitoring signals and the advantages of new modernized signals and constellations for scintillation monitoring can be found in [12]. Moreover, an interested reader can find useful material about the design of monitoring stations in [6,13,14,15,16] and alternative receiver architectures for monitoring of ionospheric scintillations by means of GNSS signals in [17,18].

With the evolving of the artificial intelligence world, machine learning (ML) algorithms started to be applied also for scintillation detection. One of the proposed methods is based on a support vector machines (SVM) algorithm [19] that belongs to the class of supervised machine learning algorithms [20]. Supervised algorithms require large data sets to properly train the algorithm so that it is able to recognize the scintillation presence during the analysis of new measurements. In [21], another type of supervised learning algorithm, namely, the decision tree, is applied to enable early scintillation alerts. In [22], the performance of SVM implementations for phase and amplitude scintillation detection have been evaluated. The main weakness of these works is that the impact of the design parameters of the different algorithms on the achieved performance has not been carefully analyzed in detail as witnessed by the limited literature addressing the application of machine learning to GNSS. Since the success of the SVM algorithm can be attributed to the joint use of a robust classification procedure and of a versatile way of pre-processing the data, the parameters of the machine learning phase must be carefully chosen [23].

The SVM algorithm is the most widely used kernel learning algorithm [19]. The kernel method enables the SVM algorithm to find a hyperplane in the kernel space by mapping the data from the feature space into higher dimensional kernel space and leading to achievement of nonlinear separation in the kernel space [24]. Kernel representations offer an alternative solution to increase the computational power of the linear learning machines [25]. In SVM implementations, the kernel functions are linear, Gaussian radial basis function (RBF), and polynomial are widely used. Hence, the problem of choosing an architecture for an ML-based application is equivalent to the problem of choosing a suitable kernel for an SVM implementation [25]. Moreover, when training an SVM algorithm, besides choosing a suitable kernel function, a number of decisions should also be made in the preparation of the data, by labeling them, and setting the parameters of the SVM [26]. Otherwise, uninformed choices might result in degraded performance [27].

In this paper, we extend our previous work [28] which involves the performance comparison of linear and Gaussian kernels for phase scintillation detection versus the analysis of both amplitude and phase scintillation events. Linear and Gaussian kernels, the implementation of different order polynomial kernels, and the performance comparison on the cross-validation results are the original core of this paper. The impact of the kernel function on the scintillation detection performance by considering the related design parameters (e.g., scale parameter, polynomial order) is discussed. Performance is assessed by exploiting the receiver operating characteristics (ROC) curves, confusion matrix results and the related performance metrics. This study is performed using real GNSS signals that are affected by significant phase and amplitude scintillation effect, collected at the South African Antarctic research base (SANAE IV, $71.67^{\circ }$ S, $2.84^{\circ }$ W) and Vietnam (Hanoi, $21.00^{\circ }$ N, $105.85^{\circ }$ E).

The paper is organized as in the following. In Section 2, we introduce the scintillation measurements and the analysis of the real data. In Section 3, we provide an overview of the SVM algorithm. Then, implementation and performance analysis of the SVM algorithm with different kernel functions for scintillation detection and the comparison of the test results are discussed in Section 4. Finally, Section 5 draws conclusions.

## 2. Ionospheric Scintillation Data and Analysis

The amount of amplitude and phase scintillation that affect the GNSS signal can be monitored and measured by exploiting the signal tracking stage correlator outputs in the GNSS receiver. Specialized Ionospheric Scintillation Monitoring Receivers (ISMRs) or software defined radio (SDR) based receivers can be used for monitoring purposes [6,13,14,15,16,29].

**Figure 1:**
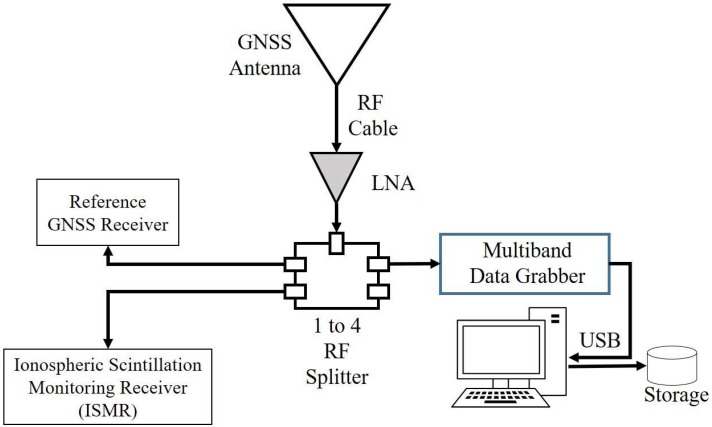
Experimental scintillated GNSS data collection configuration.

Figure 1 shows an example of scintillation monitoring and data collection setup. A part of the data that are used throughout this paper were collected in the Antarctic station and in an Equatorial site by means of data grabbers of this kind. The data-collecting setup is a custom-designed solution based on a multi-constellation and multi-frequency GNSS data grabber and a software-defined radio receiver [15,30]. Data sets acquired in Antarctica grow constantly, and several levels of the data availability such as public access, limited access, and data license are offered [30].

Fourtune, which is the type of multiband data grabber shown in Figure 1, is a multi-frequency data collection unit, and it is able to perform medium-complexity signal processing (e.g., decimation, digital filtering, quantization, etc.). It has been developed by the researchers at Joint Research Center (JRC) of the European Commission in Italy and an interested user can find more information about its architecture in [16]. Scintillated data sets that are collected via Fourtune and used in this paper are summarized in Table 1. Pseudo-Random Noise (PRN) codes are ranging code components of the transmitted satellite signals and are unique for each satellite signal. In Table 1, they refer to the satellites from which the scintillation effect observed in the received signals.

Typically, there are two parameters to indicate the amount of scintillation effect. Amplitude scintillation is monitored by computing the $S_{4}$ index which corresponds to the standard deviation of the detrended signal intensity [15]. In an ISMR, $S_{4}$ metric is calculated as [16]

(1) $$S_{4}=\sqrt{\frac{{\langle I^{2}\rangle }_{T}-{\langle I\rangle }_{T}^{2}}{{\langle I\rangle }_{T}^{2}}},$$

where *I* is the detrended signal intensity, ${\langle \cdot \rangle }_{T}$ is the average operation over a fixed period *T*. Thus, as to compute the scintillation effect on the signal amplitude and to remove the variations due to other effects, detrending operations that commonly correspond to processing the signal intensity through cascaded low-pass filters are employed. On the other hand, phase scintillation monitoring is achieved by computing the $\sigma_{\phi }$ index corresponding to the standard deviation of the detrended phase measurements [16]:

(2) $$\sigma_{\phi }=\sqrt{{\langle \varphi^{2}\rangle }_{T}-{\langle \varphi \rangle }_{T}^{2}},$$

where φ is the detrended phase measurement that can be obtained by processing the carrier phase measurements through three cascaded second-order high pass filters or Butterworth high-pass filter [16]. Moreover, useful material in the design of filters for phase and amplitude detrending algorithms can be found in [11].

**Figure 2:**
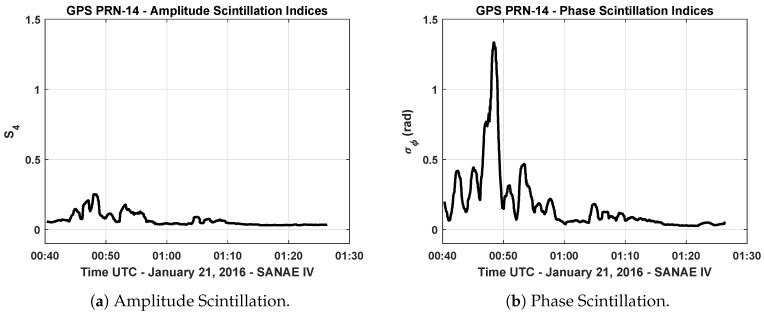
Scintillation index values of GPS L1 C/A PRN-14 signal—21 January 2016 (SANAE IV).

Figure 2a,b show an example of the computed amplitude and phase scintillation indices of GPS L1 signal that belongs to the data collected at Antarctic station on 21 January 2016. In Figure 2b, a sharp increase starting around 12:50 a.m. can be noticed, which indicates that the GPS signal broadcast from PRN-14 satellite experiences strong phase scintillation. On the other hand, in Figure 2a, there is no increase observed in the computed $S_{4}$ indices, which could be an indicator for amplitude scintillation. Due to polar location of SANAE IV, phase scintillation statistically occurs more often than amplitude scintillation [31]. Owing to the fact that the occurrence of the ionospheric scintillation depends on the several factors such as solar and geomagnetic activity, geographic location, the season of the year, and local time [15], it is not straightforward to model the occurrence of the event, and statistical analyses have been exploited in order to characterize the intensity, duration, and occurrence frequency of the scintillation events observed in different locations [31,32].

**Figure 3:**
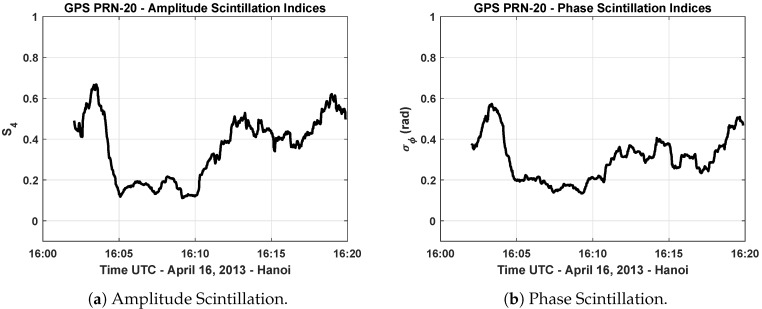
Scintillation index values of GPS L1 C/A PRN-20 signal—16 April 2013 (Hanoi).

Figure 3a,b show an example of the computed amplitude and phase scintillation indices of GPS L1 signal that is broadcast from the PRN-20 satellite. It is seen that both amplitude and phase scintillations occur at the same time in this data that were collected in Hanoi on 16 April 2013. In the scintillation events observed at the equatorial region, typically both phase and amplitude scintillations occur with faster and deeper signal power fadings that take longer durations [32].

After having analyzed the scintillated data, the detection of the scintillation and the test results will be explained in the following sections.

## 3. Overview of Support Vector Machines Algorithm

**Figure 4:**
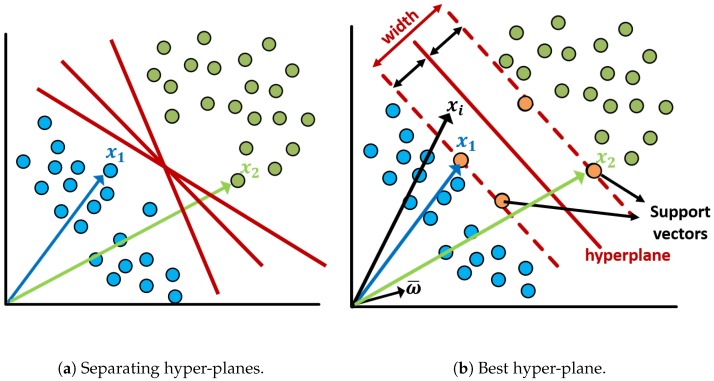
An overview sketch of Support Vector Machines (SVM) algorithm linear classifier.

An SVM algorithm classifies the data by finding the best hyper-plane that separates all the data of one class from those of the other class [33]. Figure 4 shows a pictorial example of two data sets that can be separable into two classes. However, as it can be seen in Figure 4a, there could be an infinite number of separating hyper-planes. The classes can be separated by the linear boundaries as well as nonlinear boundaries. SVM approaches this problem through the concept of the smallest distance between the decision boundary and any of the data samples [34]. In Figure 4b, as an example of linear classifiers, the best hyper-plane that corresponds to the one providing the largest margin between the classes is depicted. The margin is the maximum width of the slice, parallel to the hyper-plane that has no data samples within [33]. The data samples that are closest to the separating hyper-plane are called support vectors as shown in Figure 4b.

In SVM linear classification, the idea is to take the projection of an unknown vector $x_{i}$ along vector $\bar{\omega }$, which has to be perpendicular to the decision boundary medium (e.g., it has to be in the third dimension if the decision boundary is spanned in two dimensions), and to check whether it crosses the boundary or not in order to decide the classification. The implementation starts with the derivation of the optimal hyper-plane of SVM.

### 3.1. Derivation of the Optimum Hyper-Plane

With the given input data-set $\left\{x_{1},x_{2},...,x_{N}\right\}$ where $x_{i}\in R^{n}$, the two-class data classification problem using linear models are written in the following form [34]:

(3) $$y_{i}=f\left(x_{i}\right)=\omega_{0}^{T}x_{i}+b_{0},$$

where $\omega_{0}^{T}x$ corresponds to the projection of *x* over the direction spanned by $\omega_{0}$. $\omega_{0}$ and $b_{0}$ are parameters of the optimum hyperplane [20]. Corresponding target values $\left\{t_{1},t_{2},...,t_{N}\right\}$ to the input values are decided according to $y_{i}$:

(4) $$class\left(x_{i}\right)=\left\{\begin{array}{cccc} C_{1}, & \omega_{0}^{T}x_{i}\geq -b_{0} & \to & t_{i}=+1,\\ C_{2}, & \omega_{0}^{T}x_{i}<-b_{0} & \to & t_{i}=-1, \end{array}\right.$$

where $t_{i}$∈$\left\{-1,+1\right\}$ defines the class labels. If all the points are classified correctly, it leads to $t_{i}y_{i}>0$ for all *i*. The distance of $x_{i}$ from the hyperplane is computed as [35]

(5) $$d\left(x_{i}\right)=\frac{\left|f(x_{i})\right|}{∥\omega ∥}=\frac{t_{i}\left(\omega_{0}^{T}x_{i}+b_{0}\right)}{∥\omega ∥}.$$

Up to this point, there are not enough constraints to fix specific values for *b* and ω. Therefore, in order to define a decision rule and have an optimum hyper-plane, the parameters should be optimized by considering the maximum width for the margin that can be obtained by solving [34]

(6) $$\underset{\omega_{0},b_{0}}{arg max}=\left\{\frac{1}{∥\omega ∥}\min_{i}\left[t_{i}\left(w^{T}x_{i}+b\right)\right]\right\}.$$

The direct optimization problem would be very complex and this constrained optimization problem is solved by using Lagrange Multipliers [34]. The cost function is written as

(7) $$L=\frac{1}{2}{∥\omega ∥}^{2}-\sum_{i=1}^{N}a_{i}\left\{t_{i}\left(\omega^{T}x_{i}+b\right)-1\right\},$$

where $a={\left(a_{1},a_{2},...a_{N}\right)}^{T}$ are Lagrangian multipliers with $a_{i}\geq 0$. The solutions are saddle points of the cost function *L*. There is a minus sign before the multipliers because saddle points are obtained by minimizing with respect to ω and *b* and maximizing with respect to *a* [20,34]. The derivatives of the cost function with respect to ω and *b* are then set to zero:

(8) $$\frac{\partial L}{\partial \omega }=0\to \omega_{0}=\sum_{i=1}^{N}\alpha_{i}t_{i}x_{i},$$

(9) $$\frac{\partial L}{\partial b}=0\to \sum_{i=1}^{N}\alpha_{i}t_{i}=0.$$

Replacing Equations (8) and (9) in Equation (7) leads to the dual representation of the maximum margin problem [34]

(10) $$\tilde{L}\left(a\right)=\sum_{i=1}^{N}a_{i}-\frac{1}{2}\sum_{i=1}^{N}\sum_{j=1}^{N}a_{i}a_{j}t_{i}t_{j}\kappa \left(x_{i},x_{j}\right)\;\;\;\;\mathrm{subject}\;\mathrm{to}$$

(11) $$a_{i}\geq 0,\;\;\;i=1,...,N,$$

where $\kappa \left(x_{i},x_{j}\right)=x_{i}^{T}x_{j}$ is kernel function. A necessary and sufficient condition for a function $\kappa (x_{i},x_{j})$ to be a valid kernel, the function $\kappa (x_{i},x_{j})$ should be positive semi-definite for all the possible choices, and this also ensures that the Lagrangian function $\tilde{L}\left(a\right)$ is bounded [34]. The Lagrange multipliers are obtained by solving

(12) $$\hat{a}=\underset{a}{arg max}\tilde{L}\left(a\right),$$

with respect to a subject to the constraints

(13) $$\hat{a}\geq 0,i=1,2,...,N,$$

(14) $$\sum_{i=1}^{N}{\hat{a}}_{i}ti=0,$$

where all the points that have $\hat{a}\neq 0$ are on the margin; in other words, they are support vectors [24]. For all the points that do not lie on the margin, the corresponding Lagrange multiplier is $\hat{a}=0$. Now, $y_{i}$, defined by Equation (3), can be evaluated as

(15) $$y_{i}=f\left(x_{i}\right)=\sum_{j=1}^{N}{\hat{a}}_{j}t_{j}\kappa \left(x_{i},x_{j}\right)+b.$$

The optimization of this form satisfies the following Karush–Kuhn–Tucker (KKT) conditions that are both necessary and sufficient for optimum SVM solution [33,34,35]:

(16) $$\begin{array}{cc} {\hat{\alpha }}_{i} & \geq 0,\;\;\forall i,\\ t_{i}y_{i}-1 & \geq 0,\;\;\forall i,\\ {\hat{\alpha }}_{i}\left\{t_{i}y_{i}-1\right\} & =0,\;\;\forall i. \end{array}$$

After obtaining the Lagrange multipliers $\hat{a}$ by solving Equation (12), the optimal parameters $w_{0}$ and $b_{0}$ can be computed by considering the set of support vectors *S* because any data point for which $\hat{a}=0$ plays no role in making predictions for the new data points. Having obtained the $\hat{a}$ and support vector $x_{i}$ values that satisfy KKT conditions and Equation (15), the value of the threshold parameter *b* can be determined [34]

(17) $$w_{0}=\sum_{i\epsilon S}{\hat{a}}_{i}t_{i}x_{i},$$

and

(18) $$b_{0}=\frac{1}{N_{S}}\sum_{i\epsilon S}\left(t_{i}-\sum_{j\epsilon S}{\hat{a}}_{j}t_{j}\kappa \left(x_{i},x_{j}\right)\right),$$

where $N_{S}$ is the total number of support vectors in the set *S* and averaging over all support vectors provides a numerically more stable solution for $b_{0}$ [34]. Eventually, the decision function of SVM is computed as

(19) $$\hat{y}\left(x_{i}\right)=\mathrm{sign}\left(\sum_{j=1}^{N_{s}}\left[{\hat{a}}_{i}t_{i}\kappa \left(x_{j},x_{i}\right)\right]+b_{0}\right).$$

### 3.2. Kernel Extension

A kernel is a function κ that for all x,y∈X satisfies [36]

(20) $$\kappa \left(x,y\right)=\langle \phi (x),\phi (y)\rangle ,$$

where ϕ is a mapping from *X* to $F_{\kappa }$ that is an inner product feature space associated with the kernel κ:

(21) $$\phi :x\in X\to \phi \left(x\right)=\kappa \left(x,\cdot \right)\in F_{\kappa }.$$

Any finite subset of the space *X* is positive semi-definite, and the kernel function satisfies a positive semi-definite condition as mentioned. Actually, corresponding space $F_{\kappa }$ is referred to as Reproducing Kernel Hilbert Space (RKHS), which is a Hilbert space containing the Cauchy sequence limit condition [36]. The theory of reproducing kernels was published by Aronszajn in 1950, and detailed theory can be found in [37]. Moreover, the kernel concept was introduced into the pattern recognition field by Aizerman in 1964 [34].

In most cases, the samples may not be linearly separable. As it is seen in Equation (10), linear kernel can be expressed as

(22) $$\kappa \left(x_{i},x_{j}\right)=x_{i}^{T}x_{j}.$$

If the classification problem is not linearly separable, SVM can be powered up by a proper kernel function. The kernel method enables SVM to find a hyperplane in the kernel space and hence nonlinear separation can be achieved in that feature space [24]. An example of nonlinear kernels is Gaussian RBF, which can be written as

(23) $$\begin{array}{cc} \kappa (x_{i},x_{j}) & =\exp\left(-\frac{{∥x_{i}-x_{j}∥}^{2}}{2\sigma^{2}}\right)\\ \; & =\exp\left(-\gamma {∥x_{i}-x_{j}∥}^{2}\right), \end{array}$$

where σ defines the width of the kernel. If the parameter σ is close to zero, SVM tends to over-fitting, which means all the training instances are used as support vectors [24]. Assigning a bigger value to σ may cause under-fitting, leading all the instances to be classified into one class. Therefore, a proper value must be selected for the kernel width. In the same manner, kernel scale parameter corresponds to γ parameter in the RBF definition as being different from the σ representation.

Another most commonly used kernel function is the polynomial that can be represented as

(24) $$\kappa \left(x_{i},x_{j}\right)={\left(1+x_{i}^{T}x_{j}\right)}^{p},$$

where *p* is the order of the polynomial kernel. The lowest degree polynomial corresponds to the linear kernel, and it is not preferred in case of having a nonlinear relationship between the features. The degree of the polynomial kernel controls the flexibility of the classifier and higher-degree allows a more flexible decision boundary compared to linear boundaries [38].

The performance of linear, Gaussian, and polynomial kernels are compared in our implementation in the next section.

## 4. Experimental Tests

In this section of the paper, the implementation and performance analysis of the scintillation detection based on the SVM method that exploits different kernel functions are provided through the collected data.

### 4.1. Training Data Preparation and Labeling

The preparation of the data is the most important step in the machine-learning implementations. Amplitude and phase scintillation indices have to be put into a format so that the SVM algorithm can detect the scintillation in the correct way. As it is mentioned, it is difficult to model the occurrence of scintillation due to temporal and spatial variabilities of the ionosphere [32]. Statistical analysis has been highly benefited and also chosen fixed period (*T*) for the computations of $S_{4}$ and $\sigma_{\phi }$ indices is quite important, given in Equations (1) and (2). Generally, *T* is adjusted to 60 s in ISMR receivers. In this case, by considering only one value to feed the algorithm, early detection of the scintillation seems not to be possible. Moreover, according to performed analysis of high-latitude and equatorial ionospheric scintillation events in [32], phase scintillation lasts around 5.6 min at high latitude regions and 10.2 min in the equatorial region. On the other hand, it has been observed that amplitude scintillation events last around 3.1 min at the high-latitude region and 12.4 min in the equatorial region. Therefore, so as to enable early scintillation detection, the training data are put into a format by partitioning the data into three-minutes blocks via a moving time window.

In this work, only two class labels, namely, scintillation and no-scintillation, are assigned as follows:

(25) $$class\left(x_{i}\right)=\left\{\begin{array}{ccc} C_{1}\; & \left\{\mathrm{Scintillation}\right\} & ,t_{i}=1\;\left(\sigma_{\phi }\geq 0.3\;\;\mathrm{or}\;\;S_{4}\geq 0.4\right),\\ C_{2}\; & \left\{\mathrm{No}-\mathrm{Scintillation}\right\} & ,t_{i}=0\;\left(\sigma_{\phi }<0.3\;\;\mathrm{or}\;\;S_{4}<0.4\right). \end{array}\right.$$

**Figure 5:**
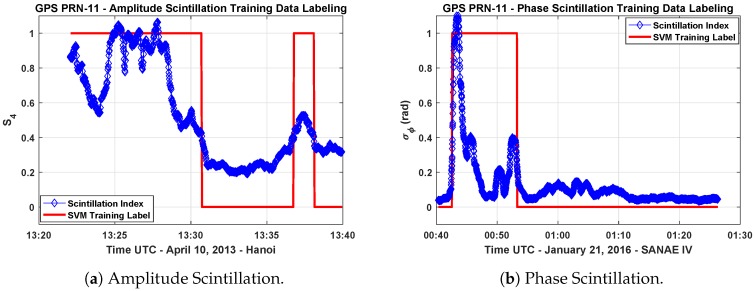
Labeling of the amplitude and phase scintillation index values in the training data sets.

The class definitions in Equation (25) have been decided according to the limit values of $\sigma_{\phi }$ and $S_{4}$ indices given in [39,40] that are observed in high-latitude and equatorial scintillation events. The list of the training data segments is reported in Table 1. The data collected on different days at SANAE IV and Hanoi stations are put in the defined structure and labeled according to the class definition above. Figure 5a,b show an example of labeling for both amplitude and phase scintillation data sets.

In Figure 5a, $S_{4}$ indices computed from the received GPS L1 signal broadcast from the satellite PRN-11 are plotted. It belongs to the data collected on 10 April 2013 in Hanoi. The figure indicates that amplitude scintillation events occurred starting around 1:20 p.m.

Another event that is observed in the data collected on 21 January 2016 at the Antarctic station SANAE IV is analyzed, and the computed $\sigma_{\phi }$ indices are plotted in Figure 5b. The figure shows the phase scintillation event occurred starting from 12:40 a.m. as denoted by the sharp increase in the indices. Although the $\sigma_{\phi }$ values go to values lower than 0.3 around 12:50 a.m., such a time interval is still considered to be part of the scintillation event and then labeled accordingly. In fact, the data portion between the consecutive scintillation events is affected anyway by a residual scintillation effect that can be observed in the receiver tracking outputs and GNSS measurement observables. Therefore, labeling has been done manually, by inspection, for all the training data sets.

### 4.2. Cross Validation

After class labels are assigned to the data sets for amplitude and phase scintillation events, SVM methods with different kernel functions are trained. In this section, the performance of validation of the methods is evaluated in terms of ROC curves.

**Figure 6:**
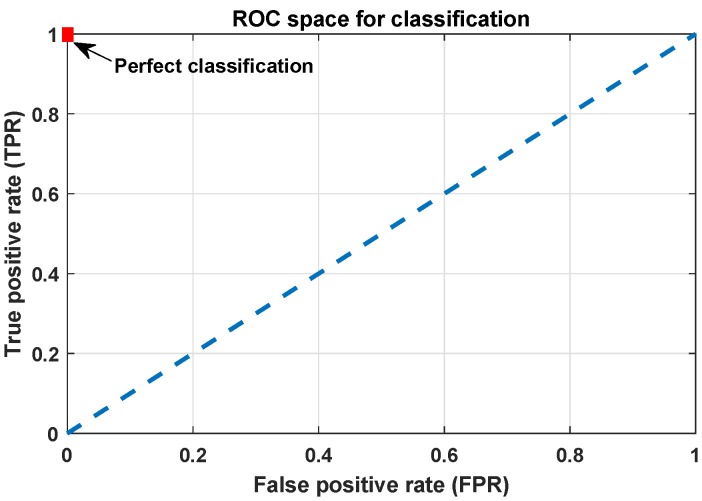
An example of Receiver Operating Characteristics (ROC) space for classification evaluation.

Figure 6 shows an example of the ROC graph. It is a two-dimensional plot of a classifier indexed in one dimension by the false positive rate (FPR) and in the other by the true positive rate (TPR). An ROC graph depicts relative trade-offs that a classifier makes between benefits (true positives) and costs (false positives) [41]. True positive rate (i.e., sensitivity) and the true negative rate (i.e., specificity) are the terms that split the predictive performance of the classifier into the proportion of positives and negatives correctly classified, respectively.

In ROC space, the point (0,1) represents the perfect classification as shown in Figure 6. The dashed diagonal line represents the case of random assignment of an element to a class. If one point is closer to the upper left corner (i.e., higher TPR, lower FPR), its classification performance is better than another. Classifiers appearing on the left side of the ROC space are called conservative such that they make classification with strong evidence so they have small FPR, but they generally have low TPR [42]. On the other hand, the classifiers on the right side of the ROC space are thought as liberal. They make classifications with low evidence so they classify all the positives correctly with a drawback of high FPR. Each point on the ROC curve represents a trade-off, in other words, a cost ratio [43]. Cost ratio is equal to the slope of the line tangent to the ROC curve at a given point.

SVM implementation for both phase and amplitude scintillation detection has been done by employing MATLAB’s statistics and machine learning toolbox (R2016b, The Mathworks, Inc., Natick, MA, USA) [44]. In order to evaluate the performance of the implemented SVM methods, a 10-fold cross-validation technique has been applied. In this technique, the partitions are put into 10 randomly chosen subsets of equal size. Then, each subset is used to validate the model by using the trained remaining nine subsets. This process is repeated 10 times so that each subset is used once for the validation.

**Figure 7:**
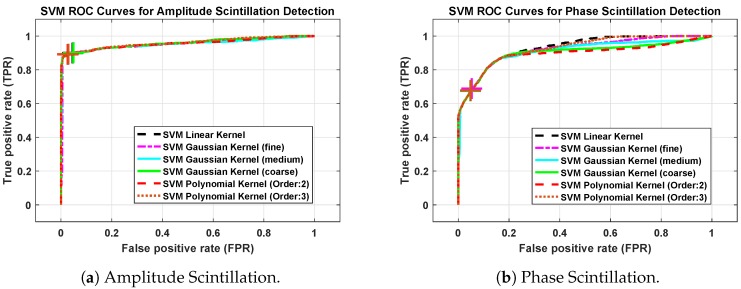
Receiver Operating Characteristics (ROC) curves of Support Vector Machines (SVM) methods with different kernel functions.

Figure 7a,b show the ROC curves of SVM methods with different kernel functions for amplitude and phase scintillation, respectively. In general, it shows that, in an amplitude scintillation case, the performance of the SVM methods is better than the phase scintillation case. However, due to the fact that SVM performance depends on the data sets, we evaluate each scintillation case separately and compare the performance of the kernel functions under the same conditions.

In the analysis, Gaussian RBF kernel scale parameter γ in Equation (23) is adjusted to different values according to the following assumptions [45]:

(26) $$\begin{array}{cc} \gamma_{fG} & =\sqrt{n/4},\;\;\mathrm{for}\;\mathrm{fine}\;\mathrm{Gaussian},\\ \gamma_{mG} & =\sqrt{n},\;\;\mathrm{for}\;\mathrm{medium}\;\mathrm{Gaussian},\\ \gamma_{cG} & =4\sqrt{n},\;\;\mathrm{for}\;\mathrm{coarse}\;\mathrm{Gaussian}, \end{array}$$

where *n* is the number of features or the dimension size of $x_{i}$ in Equation (3). Moreover, second and third-order polynomial kernels are included in the analysis. The different colored plus symbols on the figures indicate the operating points of each method in Figure 7. Since the ROC curves are close to each other and it is not easy to differentiate the differences between each method, the results are summarized in Table 2 and Table 3.

In Table 2 and Table 3, we compare the time complexity values that are dedicated time to both training and testing. The complexity of a classifier is divided into two kinds of complexity, namely, time complexity and space complexity [46]. While time complexity deals with the time spent on the execution of the algorithm, space complexity considers the amount of memory used by the algorithm [46]. For example, run-time complexity of linear and RBF kernels differ from each other. While the complexity of the RBF kernel is shown to be $O\left(n_{SV}\times d\right)$, which is dependent on the number of support vectors ($n_{SV}$) and the input dimension (*d*), linear method has $O\left(d\right)$ prediction complexity [47]. Therefore, as it is seen in both Table 2 and Table 3, running time increases in the cases of the kernel functions in which the samples are uplifted into higher dimensions, and it also becomes dependent on the number of samples.

The parameter, Area Under Curve (AUC), represents the estimated area under the ROC curve, and it is used as a peformance measure for the machine learning algorithms [48]. AUC is accepted as an indicator for the overall accuracy of the classifier; both Table 2 and Table 3 show the importance of kernel scale parameter. While the overall accuracy of coarse and medium Gaussian kernel SVM methods are less than the linear SVM, fine Gaussian SVM outperforms the linear. Moreover, the third-order polynomial kernel provides improved performance compared to linear, coarse, and medium Gaussian kernel SVMs, but it comes with a cost of increased complexity and time.

### 4.3. Tests and Evaluation

**Figure 8:**
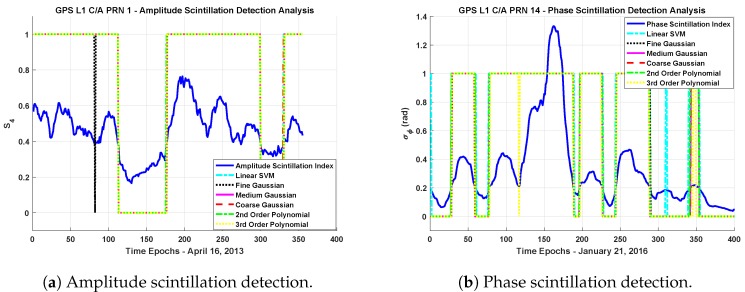
Scintillation detection results based on Support Vector Machines (SVM) with different kernel functions. “1” corresponds to the points in which the related method points out the scintillation event and “0” means no-scintillation event is detected. Both amplitude and phase scintillation indices synchronized in time can be evaluated as ground truth in the graph to evaluate the performances of different kernels.

In this section of the paper, as a performance cross-check, we also used the collected data which are not included in the training sets to evaluate the performance of scintillation detection methods. Figure 8a,b show the decisions of the different kernel SVM methods for the data sets for both amplitude and phase scintillation.

In order to evaluate the performance comparison, the confusion matrix technique, which is a two-dimensional matrix indexed in one dimension by the true class of an object and in the other by the class that the classifier assigns [49] as it is seen in Table 4, is applied. A confusion matrix represents the dispositions of a set of instances (i.e., test data) according to a defined classification model that maps the set of instances to predicted classes [42]. Actually, each different point in a ROC curve corresponds to a confusion matrix.

In the two-class data classification problem, the four cells of the confusion matrix correspond to the values of true positives (TP), false positives (FP), true negatives (TN), and false negatives (FN). By considering the numbers of the measures, this matrix forms the basis for the terms, namely, accuracy, precision, sensitivity, specificity, and error rate. Accuracy is the ratio of the correct predictions (i.e., the sum of true positives and true negatives) to the total predictions made by the classifier. In the same sense, the error rate is the ratio of the incorrectly classified objects to the total objects. In Table 5, the performance of different kernels for the data sets in terms of accuracy and error rate are summarized.

As it is expected, the results in Table 5 are consistent with the cross-validation results. With the correct setting of the kernel scale parameter, the fine Gaussian SVM method outperforms in terms of accuracy rate. Furthermore, although the differences in the accuracy of the methods seem not to be at a considerable level, in terms of early detection, the method that provides higher accuracy gains importance. For example, in the cases of the computation rate of the scintillation indices being around one minute, the method having a higher accuracy rate will provide quite advantageous conditions in terms of early detection. However, the third order polynomial kernel provides an improvement in the accuracy compared to the linear kernel, but its performance should be evaluated with increased time and space complexity.

## 5. Conclusions

In this paper, we provided performance analysis comparing different kernel methods of SVM and we reviewed and analyzed the linear, Gaussian, and polynomial kernel SVM algorithms for both phase and amplitude scintillation detection. Performance comparison was assessed by exploiting the ROC curves, confusion matrix results, and the performance metrics associated with the confusion matrix. It has been observed that, if the kernel scale parameter of Gaussian RBF kernel SVM algorithm is optimized, the performance of the RBF kernel SVM method outperforms the linear kernel SVM method in terms of overall accuracy. Moreover, although third order polynomial kernel SVM performs better than the linear kernel, it comes with a cost of increased time and space complexity.

For a possible scintillation detection implementation that could be based on unsupervised algorithms in the future, the effect of the multipath has to be considered because the presence of the multipath causes an increase in the amplitude scintillation index values indicating scintillation alarm falsely.

## Figures and Tables

**Table 1:** Specifications of the scintillated data sets with visible Pseudo-Random Noise (PRN) codes and location of the stations.

	Dates	PRNs	Station	Coordinates
1	21 January 2016 3 February 2016 8 February 2016 17 August 2016	11,14,22 3,6 1,12,14 9	South African Antarctic Research Base (SANAE-IV), Antarctic	Lat.: 71.67278° S Long.: 2.840556° W
2	10 April 2013 12 April 2013 16 April 2013 4 October 2013	11 17 1,20,28 15,21,24	Hanoi, Vietnam	Lat.: 21.004556° N Long.: 105.843917° E

**Table 2:** Phase scintillation detection performance comparison in terms of complexity, True Positive Rate (TPR), False Positive Rate (FPR), and Area Under Curve (AUC) under 10-fold cross-validation test.

SVM Method	Kernel Scale	Running Time	Validation Accuracy (%)	Operating Point		AUC (%)
				TPR	FPR	
Linear	1	$t_{p}$	86.01	0.6772	0.0468	91.98
Coarse Gaussian	6.9	1.28$t_{p}$	86.29	0.6755	0.0482	90.10
Medium Gaussian	1.7	1.55$t_{p}$	86.16	0.6751	0.0480	90.85
Fine Gaussian	0.43	1.70$t_{p}$	85.95	0.6890	0.0530	93.16
Polynomial (Order:2)	1	1.37$t_{p}$	86.26	0.6768	0.0485	89.38
Polynomial (Order:3)	1	3.20$t_{p}$	86.04	0.6779	0.0488	92.67

**Table 3:** Amplitude scintillation detection performance comparison in terms of complexity, True Positive Rate (TPR), False Positive Rate (FPR), and Area Under Curve (AUC) under 10-fold cross-validation test.

SVM Method	Kernel Scale	Running Time	Validation Accuracy (%)	Operating Point		AUC (%)
				TPR	FPR	
Linear	1	$t_{a}$	90.44	0.8990	0.0460	95.37
Coarse Gaussian	6.9	1.02$t_{a}$	90.72	0.9004	0.0487	95.18
Medium Gaussian	1.7	1.18$t_{a}$	91.65	0.9004	0.0487	95.19
Fine Gaussian	0.43	1.33$t_{a}$	91.56	0.9018	0.0378	96.01
Polynomial (Order:2)	1	1.08$t_{a}$	91.42	0.8920	0.0265	95.61
Polynomial (Order:3)	1	1.80$t_{a}$	91.56	0.8927	0.0292	95.88

**Table 4:** Confusion matrix.

CONFUSION MATRIX		ACTUAL	
		Scintillation	No-Scintillation
PREDICTION	Scintillation	True Positive (TP)	False Positive (FP)
	No-Scintillation	False Negative (FN)	True Negative (TN)

**Table 5:** Accuracy and error rate performances of different kernel SVMs for scintillation detection

	Phase Scintillation		Amplitude Scintillation	
	**Accuracy**	**Error Rate**	**Accuracy**	**Error Rate**
Linear	90.98%	9.02%	94.46%	5.54%
Coarse Gaussian	92.61%	7.39%	94.18%	5.82%
Medium Gaussian	92.61%	7.39%	94.18%	5.82%
Fine Gaussian	92.50%	7.50%	95.02%	4.98%
Polynomial (Order:2)	92.28%	7.72%	94.46%	5.54%
Polynomial (Order:3)	93.59%	6.41%	94.74%	5.26%

## Abbreviations

GNSS	Global Navigation Satellite Systems
SVM	Support Vector Machines
ROC	Receiver Operating Characteristics
RBF	Radial Basis Function
TP	True Positive
FP	False Positive
TN	True Negative
FN	False Negative
TPR	True Positive Rate
FPR	False Positive Rate
AUC	Area Under the Curve
